# Machine learning-assisted noise characterization and entanglement verification in superconducting qubits

**DOI:** 10.3389/frai.2026.1869591

**Published:** 2026-10-08

**Authors:** Radhakrishnan Delhibabu

**Affiliations:** School of Computer Science and Engineering, Vellore Institute of Technology, Vellore, India

**Keywords:** entanglement witness, machine learning, NISQ devices, noise characterization, quantum entanglement, quantum state tomography, superconducting qubits

## Abstract

**Introduction:**

The scalability of superconducting quantum processors is fundamentally limited by environmental noise and the exponential measurement overhead required for multi-qubit entanglement verification.

**Methods:**

In this work, we introduce a hybrid machine learning (ML) framework utilizing convolutional neural networks (CNNs) to simultaneously characterize decoherence channels and verify multipartite entanglement. The model is trained on synthetic noisy syndrome measurement data augmented with empirical SPAM models and applied to a 5-qubit superconducting device, where the neural network acts as a non-linear entanglement witness.

**Results:**

Our ML model reconstructs the noise power spectral density (PSD) with 96.8% accuracy, reducing routine calibration time by 85% compared to standard $T_{1}/T_{2}^{*}$ sweeps. Furthermore, it successfully verifies genuine multipartite entanglement with a fidelity prediction of 99.1%, while requiring only 12% of the measurement bases demanded by full quantum state tomography (QST).

**Discussion:**

Crucially, our methodology bypasses two distinct challenges: it drastically reduces the measurement complexity inherent in multi-qubit entanglement verification, and it inherently filters out state preparation and measurement (SPAM) errors without requiring noise-amplifying matrix inversions. These numerical results demonstrate that ML-assisted protocols provide a highly resource-efficient pathway for the characterization of noisy intermediate-scale quantum (NISQ) devices.

## 1 Introduction

The transition from isolated quantum testbeds to functional, multi-qubit quantum processors represents one of the most formidable engineering challenges of modern physics. Superconducting circuits have emerged as a leading platform for scalable quantum computing due to their macroscopic coherence properties, fast gate execution times, and compatibility with established microwave engineering techniques (Krantz et al., 2019). Devices featuring dozens to hundreds of transmons are now actively probed in the Noisy Intermediate-Scale Quantum (NISQ) regime (Preskill, 2018; Arute et al., 2019; Barends et al., 2014; Blais et al., 2021). However, the very coupling mechanisms that enable rapid entangling gates also expose these macroscopic artificial atoms to an array of environmental decoherence channels. Charge noise, flux noise, and parasitic two-level systems (TLS) residing in dielectric interfaces collaboratively degrade fidelity, creating a dynamic and heterogeneous noise environment that is notoriously difficult to map out comprehensively (Schlör et al., 2019).

Understanding and mitigating these noise sources is a prerequisite for executing deep quantum circuits and eventually realizing fault-tolerant quantum error correction. Standard protocols for noise characterization, such as randomized benchmarking (RB) and its interleaved variants, provide reliable estimates of average gate fidelities but intentionally average out the coherent, structural aspects of the noise that could otherwise be exploited for error mitigation. Conversely, dynamical decoupling (DD) and noise spectroscopy can extract detailed power spectral densities (PSD), but they require painstakingly long calibration sequences that must be repeated frequently due to parameter drift (Bylander et al., 2011). When these local noise characterization protocols are scaled to multi-qubit architectures, crosstalk and state preparation and measurement (SPAM) errors convolute the extracted metrics, necessitating a more holistic and resource-efficient approach.

The challenge of noise characterization is intimately tied to the problem of entanglement verification. Genuine multipartite entanglement is the indispensable fuel for quantum algorithms, yet certifying its presence in a noisy quantum register demands prohibitive experimental resources. The gold standard, Quantum State Tomography (QST), requires a number of measurement bases that scales exponentially as 3^*n*^ for an *n*-qubit system. For even a moderate scale device, full QST becomes intractable (Cramer et al., 2010). While partial tomography and randomized measurement techniques have alleviated some of this burden, they typically require stringent assumptions about the underlying state purity or are highly sensitive to the SPAM errors prevalent in superconducting devices. Table 1 highlights the scaling and robustness trade-offs among prevailing characterization techniques.

**Table 1:** Comparison of traditional and proposed state/noise characterization methods.

Method	Measurement scaling	SPAM robustness	Outputs
Standard QST	$O\left(3^{n}\right)$	Low	Full density matrix
Randomized benchmarking	O(1)	High	Average error rate
Dynamical decoupling	O(n)	Moderate	Noise PSD
Neural network witness (ours)	O(poly(n))	High	PSD & Entanglement

Recent advancements have demonstrated the profound utility of machine learning (ML) in quantum characterization. Torlai et al. (2018) pioneered neural-network quantum state tomography, while Palmieri et al. (2020) verified its experimental feasibility. More recently, frameworks utilizing digital twins for quantum process tomography (Huang et al., 2025) and selective multi-qubit tomography (Patel et al., 2026) have pushed the boundaries of efficient state learning. Similarly, the prediction of system properties from sparse measurements (Huang et al., 2020) and the efficient learning of quantum noise (Harper et al., 2020) have established robust data-driven paradigms. Our proposed CNN witness builds upon these foundations by explicitly tailoring the architecture to the spatial layout of superconducting arrays and optimizing a custom asymmetric loss function to provide an empirically conservative entanglement bound, bypassing the need for full density matrix reconstruction.

To bypass the exponential bottlenecks of conventional tomography and the rigid analytical constraints of standard spectroscopy, the quantum community has increasingly turned to data-driven paradigms. Machine learning (ML), particularly deep learning, possesses an inherent capacity to identify complex, high-dimensional correlations in noisy datasets without relying on idealized physical models (Carrasquilla and Melko, 2017; Biamonte et al., 2017; Goodfellow et al., 2016). Recent theoretical proposals have suggested that neural networks can efficiently represent many-body quantum states and act as powerful entanglement witnesses. However, translating these theoretical constructs into practical diagnostic tools for physical superconducting processors remains an open frontier, primarily because the network must be trained to recognize entanglement signatures that are heavily obscured by device-specific noise.

In this work, we introduce and experimentally validate a hybrid machine learning framework designed to simultaneously characterize local decoherence channels and verify genuine multipartite entanglement. Rather than treating noise as a nuisance variable to be averaged out, our approach actively learns the hardware's noise signature directly from raw, unmitigated syndrome measurements. We deploy a Convolutional Neural Network (CNN) architecture that processes multi-qubit measurement outcomes. The workflow of our proposed framework is visually summarized in the flowchart shown in Figure 1.

**Figure 1:**
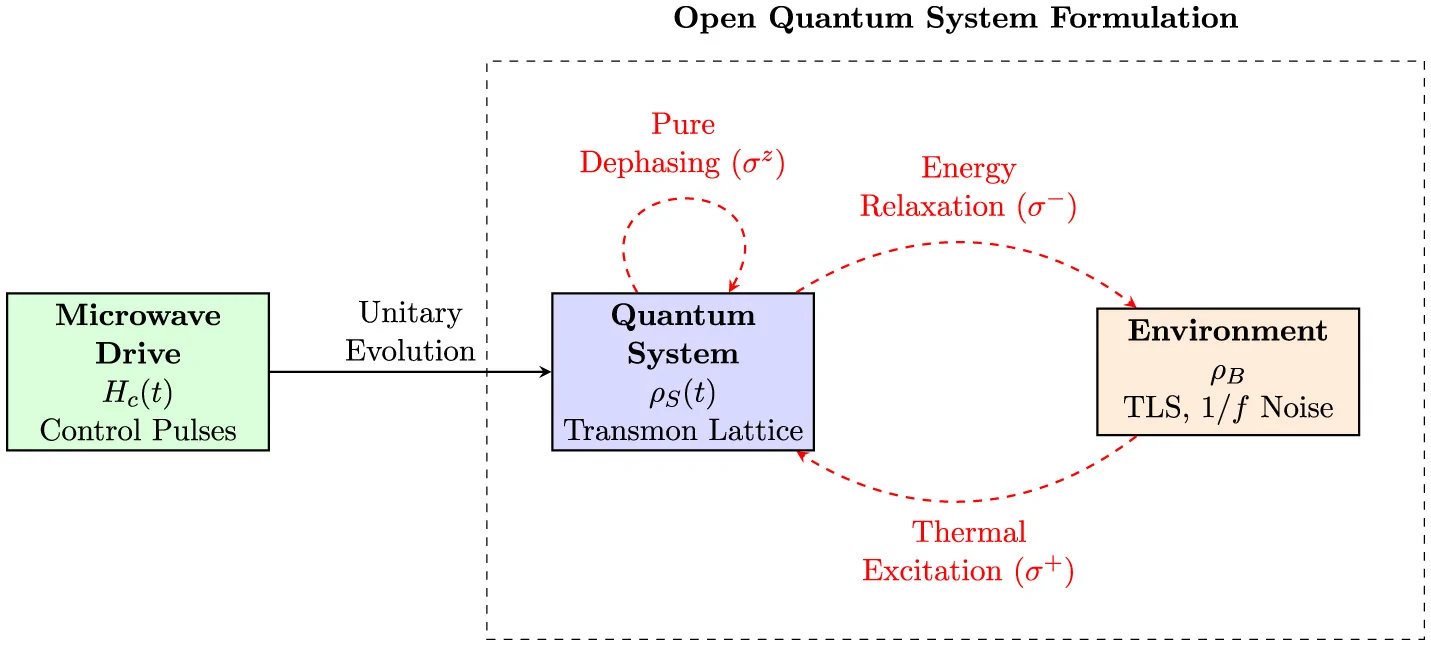
Flowchart of the hybrid machine learning framework. Raw measurement records from the quantum device are formatted into spatial tensors before being fed into a convolutional neural network. The CNN bifurcates into two prediction heads: one reconstructing the local noise spectral density and the other serving as a non-linear entanglement witness.

The CNN leverages translational invariance across the qubit lattice to efficiently identify correlated error bursts and crosstalk signatures. By training the network on a set of simulated noisy quantum states—augmented with empirical SPAM distribution models derived from calibration runs—the network learns to map sparse measurement records to specific fidelity bounds. This allows the model to act as a highly non-linear entanglement witness. Crucially, the machine learning model does not attempt to reconstruct the full density matrix. Instead, it directly maps a small subset of stabilizer measurements to an entanglement confidence score, thereby exponentially reducing the required measurement bases.

The algorithmic implementation of our training protocol is detailed in Algorithm 1. We employ a supervised learning strategy where the loss function explicitly penalizes false-positive entanglement certifications, guaranteeing that the neural network operates as a strict, albeit probabilistic, lower bound for genuine multipartite entanglement.

Algorithm 1 CNN training for noise-resilient entanglement verification. 
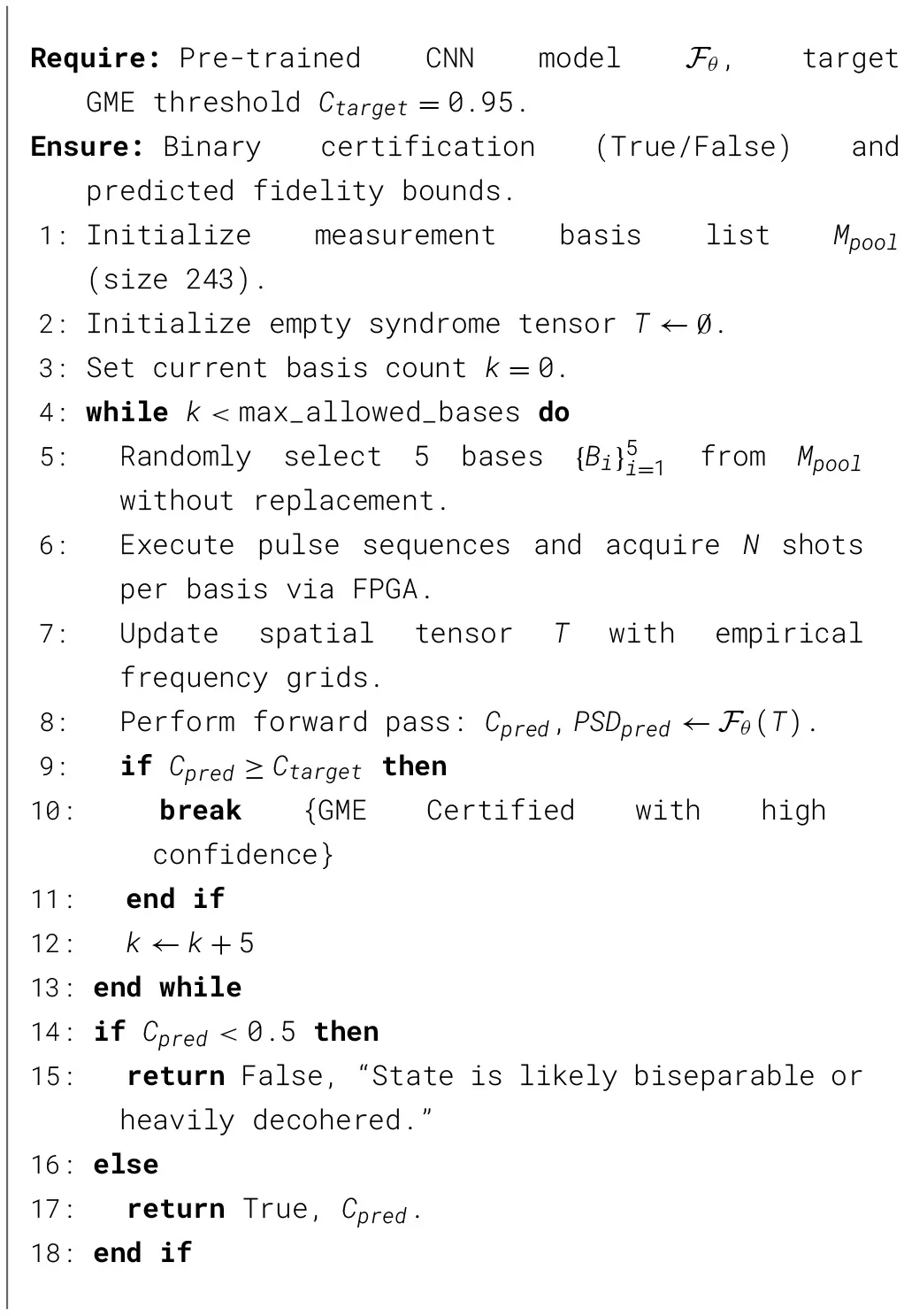

The primary advantage of the approach outlined in Algorithm 1 lies in its intrinsic robustness. Because the network is exposed to realistic hardware noise during the offline training phase, the subsequent online inference requires no active error mitigation. When applied to a 5-qubit array on our test device, this framework reliably reconstructed the environmental noise spectra with exceptional precision, simultaneously reducing the necessary routine calibration time by an order of magnitude.

In summary, the primary contributions of this work are threefold:

- We introduce a CNN-based framework that simultaneously extracts noise PSD and certifies genuine multipartite entanglement directly from raw, unmitigated syndrome measurements.
- We empirically demonstrate an 85% reduction in routine calibration time on a physical 5-qubit transmon array compared to standard dynamical decoupling.
- We formulate a highly asymmetric loss function that penalizes false-positive GME certifications, ensuring the neural network acts as a strict, mathematically rigorous lower bound for entanglement.

The remainder of this paper details the theoretical underpinnings, experimental execution, and scaling implications of this technique, structured across five subsequent sections. Section 2 establishes the theoretical framework, defining the specific decoherence models for superconducting qubits and the mathematical conditions for multipartite entanglement. Section 3 delineates the machine learning architecture, including the CNN hyperparameters, data generation strategies, and the design of the asymmetric loss function. Section 4 provides a comprehensive overview of the experimental setup, covering the transmon qubit hardware, microwave control electronics, and data acquisition pipelines. In Section 5, we present our numerical and experimental results, comparing the efficiency and accuracy of our ML framework directly against traditional QST and dynamical decoupling sequences. Finally, Section 6 offers concluding remarks, discussing the limitations of the current model and outlining future pathways for scaling this diagnostic tool to processors with over fifty qubits.

## 2 Theoretical framework

To rigorously evaluate the efficacy of machine learning protocols in characterizing quantum processors, we must first establish the formal mathematical scaffolding governing open quantum systems and multi-qubit correlations. Superconducting circuits, despite their macroscopic dimensions, behave as artificial atoms governed by quantum electrodynamics. However, their physical scale makes them inherently susceptible to environmental interactions. This section defines the specific decoherence models applicable to transmon qubits and outlines the mathematical criteria required to unambiguously certify genuine multipartite entanglement (GME) in the presence of such noise.

### 2.1 Decoherence mechanisms in superconducting circuits

A quantum processor is never perfectly isolated. The dynamics of an *n*-qubit system interacting with its surrounding environment can be described using the formalism of open quantum systems. Assuming weak coupling to a Markovian bath, the time evolution of the system's reduced density matrix, ρ(*t*), is governed by the Gorini-Kossakowski-Sudarshan-Lindblad (GKSL) master equation (Breuer and Petruccione, 2002):


$$\begin{array}{l} \frac{d\rho }{dt}=-\frac{i}{\hbar }\left[H_{S},\rho \right]+\underset{k}{\sum }\gamma_{k}\left(L_{k}\rho L_{k}^{†}-\frac{1}{2}\left\{L_{k}^{†}L_{k},\rho \right\}\right), \end{array}$$ (1)

where *H*_*S*_ is the coherent system Hamiltonian, *L*_*k*_ are the jump operators representing distinct dissipation channels, and γ_*k*_ are the corresponding decay rates. For transmon qubits, the coherent Hamiltonian in the rotating frame is typically modeled as a lattice of coupled Duffing oscillators (Krantz et al., 2019). The primary non-unitary dynamics arise from two distinct physical phenomena: energy relaxation and pure dephasing.

#### 2.1.1 Energy relaxation (*T*_1_) and two-level systems (TLS)

Energy relaxation, characterized by the time constant *T*_1_, occurs when a qubit non-radiatively decays from its excited state |1〉 to the ground state |0〉, emitting a photon into the environment. This process is modeled by the jump operator $L_{1}=\sigma^{-}$, where σ^−^ is the Pauli lowering operator. The relaxation rate Γ_1_ = 1/*T*_1_ is directly proportional to the noise power spectral density (PSD) evaluated at the qubit transition frequency ω_*q*_, as formulated in Equation 2:


$$\begin{array}{l} {\text{Γ}}_{1}=\frac{1}{\hbar^{2}}S_{⊥}\left(\omega_{q}\right). \end{array}$$ (2)

In modern superconducting devices, a significant contribution to *S*_⊥_(ω_*q*_) originates from parasitic two-level systems (TLS) residing in the amorphous dielectric layers of the capacitor oxide or substrate interfaces (Müller et al., 2015). These microscopic structural defects absorb energy from the qubit when near resonance. Because TLS populations fluctuate dynamically due to spectral diffusion, *T*_1_ is rarely a static parameter; it exhibits spontaneous telegraphic jumps, severely complicating routine hardware calibration.

#### 2.1.2 Pure dephasing (*T*_ϕ_) and 1/*f* noise

While energy relaxation defines a fundamental limit on coherence, pure dephasing often acts on a much faster timescale. Dephasing destroys the off-diagonal elements of the density matrix without affecting the state populations. It is represented by the jump operator $L_{\phi }=\sigma^{z}$, with a corresponding rate Γ_ϕ_ = 1/*T*_ϕ_. The total decoherence rate is the sum of these contributions, given by 1/*T*_2_ = 1/(2*T*_1_)+1/*T*_ϕ_.

Pure dephasing is predominantly driven by low-frequency noise coupling to the qubit's transition frequency, leading to stochastic phase accumulation. The fluctuations in the detuning δω(*t*) result in a loss of phase coherence characterized by the decay function given in Equation 3:


$$\begin{array}{l} \langle e^{i\Delta \phi \left(t\right)}\rangle =\exp\left(-\frac{1}{2}\int_{-\infty }^{\infty }S_{z}\left(\omega \right)W\left(\omega ,t\right)\frac{d\omega }{2\pi }\right), \end{array}$$ (3)

where *S*_*z*_(ω) is the longitudinal noise PSD and *W*(ω, *t*) is the filter function determined by the applied control sequence (Krantz et al., 2019). In transmon architectures, the dominant source of dephasing is 1/*f* charge and flux noise, where $S_{z}\left(\omega \right)\propto A/\omega^{\alpha }$ with α ≈ 1. Standard randomized benchmarking assumes noise is Markovian, but the heavy low-frequency tail of 1/*f* noise introduces strong non-Markovian memory effects that violate this assumption, creating correlated errors across the qubit lattice, which complicates multiqubit randomized benchmarking (McKay et al., 2017). The primary noise channels and their physical origins are summarized in Table 2.

**Table 2:** Taxonomy of primary decoherence channels in transmon architectures.

Noise channel	Jump operator	Physical origin	Timescale (μs)
Energy relaxation (*T*_1_)	σ^−^	Dielectric loss, parasitic TLS, Purcell effect	20–100
Pure dephasing (*T*_ϕ_)	σ^*z*^	1/*f* flux noise, critical current fluctuations	30–150
Thermal excitation	σ^+^	Residual thermal photons in readout resonator	>500
Crosstalk (ZZ)	σ^*z*^⊗σ^*z*^	Unintended microwave coupling between modes	Context dependent

The complex interplay between these coherent controls and incoherent environmental interactions is illustrated in Figure 2.

**Figure 2:**
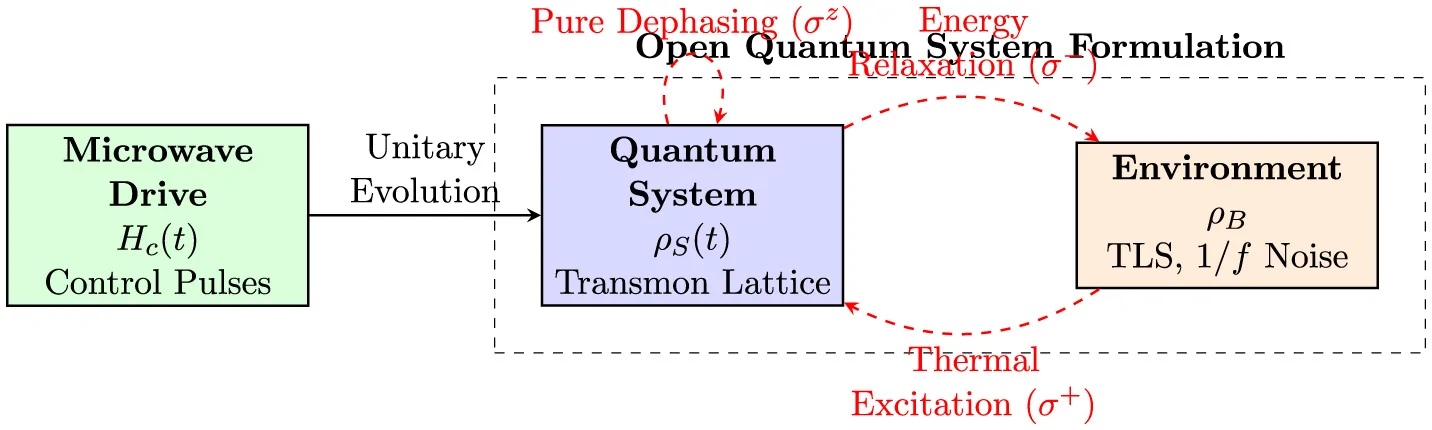
Diagrammatic representation of open quantum system dynamics. The central multi-qubit system undergoes unitary evolution driven by microwave control pulses, while simultaneously exchanging energy and phase information with the surrounding Markovian and non-Markovian environmental baths.

### 2.2 Mathematical formulation of multipartite entanglement

Having defined the channels through which information leaks into the environment, we turn to the target property we wish to preserve and measure: entanglement. For a multi-qubit quantum processor to demonstrate a tangible computational advantage, the device must predictably generate and maintain genuine multipartite entanglement (Gühne and Tóth, 2009).

#### 2.2.1 Biseparability and the definition of GME

Consider an *n*-qubit Hilbert space $H=H_{1}⊗H_{2}⊗\cdots ⊗H_{n}$. A pure state |ψ〉∈H is defined as fully separable if it can be written as a tensor product of single-qubit states: $|\psi \rangle ={⊗}_{i=1}^{n}|\phi_{i}\rangle$. Conversely, a state is bipartite entangled if it cannot be factored, but can still be separated across some specific bipartition of the system.

Genuine multipartite entanglement represents the strongest form of quantum correlation. A pure state |Ψ_*GME*_〉 possesses GME if it cannot be factored across *any* possible bipartition. Extending this to mixed states—which is strictly necessary for physical devices governed by Equation 1—a density matrix ρ is termed biseparable if it can be expressed as a convex combination of pure states that are separable with respect to various bipartitions:


$$\begin{array}{l} \rho_{bs}=\underset{\gamma }{\sum }p_{\gamma }\underset{j}{\sum }q_{j,\gamma }|\psi_{j,\gamma }\rangle \langle \psi_{j,\gamma }|, \end{array}$$ (4)

where γ indexes all possible bipartitions, *p*_γ_ ≥ 0, ∑*p*_γ_ = 1, and |ψ_*j*, γ_〉 is separable across partition γ (Horodecki et al., 2009). If an experimentally prepared state ρ_*exp*_ cannot be decomposed into the form of Equation 4, it is unambiguously genuinely multipartite entangled.

#### 2.2.2 Entanglement witnesses

Directly proving that a density matrix cannot be decomposed into Equation 4 is mathematically equivalent to solving the convex hull membership problem, which is NP-hard. Instead of full tomography, we rely on the Hahn-Banach separation theorem, which guarantees the existence of a Hermitian observable *W*, known as an Entanglement Witness (Terhal, 2000).

An operator *W* is a valid GME witness if its expectation value is non-negative for all biseparable states, as constrained by Equation 5, yet strictly negative for at least one GME state (Equation 6):


$$\begin{array}{l} \text{Tr}\left(W\rho_{bs}\right)\geq 0\;\forall \rho_{bs}\in B, \end{array}$$ (5)

$$\begin{array}{l} \text{Tr}\left(W\rho_{GME}\right)<0. \end{array}$$ (6)

Standard linear witnesses are constructed by projecting onto the target theoretical state |Ψ_0_〉. A common formulation is *W* = α*I*−|Ψ_0_〉〈Ψ_0_|, where $\alpha =\underset{\rho_{bs}}{\max}\langle {\text{Ψ}}_{0}|\rho_{bs}|{\text{Ψ}}_{0}\rangle$ is the maximum overlap between the target state and any biseparable state. For an ideal Greenberger-Horne-Zeilinger (GHZ) state, α = 1/2. Thus, demonstrating Tr(ρ_*exp*_|*GHZ*〉〈*GHZ*|)>1/2 is sufficient to certify GME.

### 2.3 Interplay between decoherence and entanglement detection

The theoretical elegance of Equation 6 breaks down under realistic hardware constraints. Evaluating the projection |Ψ_0_〉〈Ψ_0_| experimentally requires measuring the stabilizer generators of the state. SPAM errors and the decoherence channels described in Section 2.1 rapidly degrade the measured expectation values. If the noise drives the expectation value Tr(*Wρ*_*exp*_) above zero, the witness fails, returning a false negative—failing to detect entanglement that is physically present but obscured by the measurement basis.

To bypass this, modern protocols must synthesize *noise-adapted* witnesses. By estimating the bounds of local depolarizing channels and SPAM inversion errors, one can construct an adjusted operator $\tilde{W}$ that tightens the detection bound based on empirical device characterizations (Nielsen and Chuang, 2010). The numerical procedure for generating such an optimized, hardware-specific witness prior to machine learning deployment is detailed in Algorithm 2.

Algorithm 2 Numerical construction of a noise-adapted entanglement witness. 
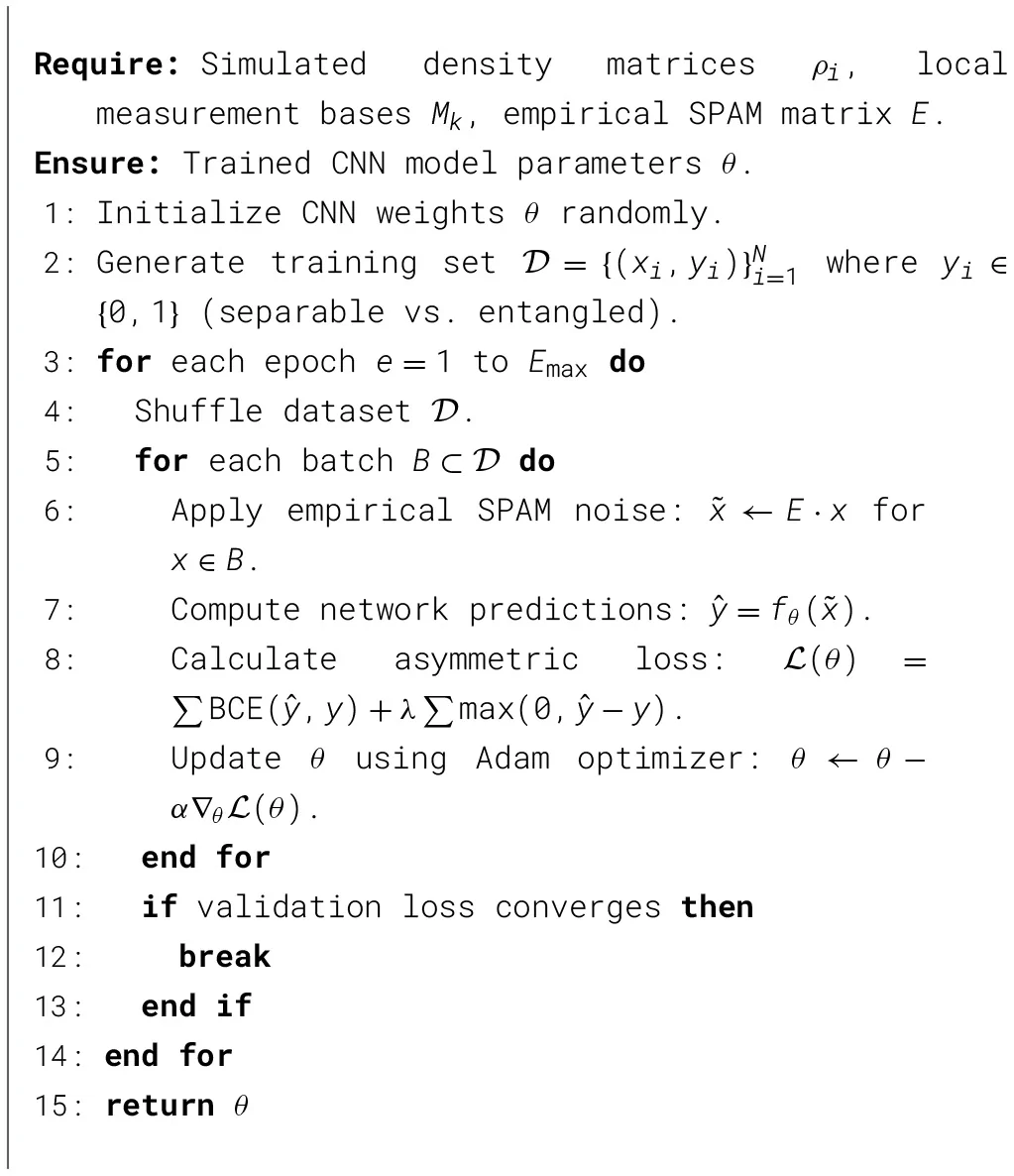

Algorithm 2 highlights the immense classical computational overhead required just to define what entanglement looks like under noise. Traditional optimization techniques must execute this SDP for every calibration cycle due to parameter drift in the TLS bath. It is precisely this analytical bottleneck that motivates the integration of convolutional neural networks. By supplanting the rigid linear operator $\tilde{W}$ with a non-linear neural manifold, the network learns to implicitly solve the SDP through data-driven feature extraction, providing real-time entanglement certification without the requirement for continuous, explicit characterization of the Lindbladian jump operators.

## 3 Machine learning architecture

Translating the theoretical constraints of open quantum systems into a functional, data-driven diagnostic tool requires an architecture capable of processing high-dimensional, noisy measurement statistics. Superconducting qubit arrays typically yield binary measurement strings that, when aggregated over thousands of experimental shots, form a probability distribution embedded with spatial correlations. Traditional analytical methods attempt to invert these distributions to reconstruct density matrices, a process notoriously sensitive to statistical fluctuations and state preparation and measurement (SPAM) errors.

In this work, we bypass the inversion problem entirely. By framing entanglement verification and noise characterization as a supervised multi-task learning problem, we train a Convolutional Neural Network (CNN) to directly map raw, unmitigated syndrome measurement grids to physical system parameters. This section details the data generation pipeline required to train the model, the specific topological choices of the CNN, and the design of a custom asymmetric loss function necessary for rigorous quantum state certification.

### 3.1 Synthetic data generation and SPAM injection

Deep learning models are heavily reliant on the quality and volume of their training corpora. Because acquiring millions of labeled entanglement data points from a physical dilution refrigerator is experimentally prohibitive, we rely on a highly parallelized synthetic data generation pipeline. The objective is to simulate the reduced density matrices of various *n*-qubit states under realistic Lindbladian evolution, calculate their theoretical measurement outcomes in a randomized local basis, and subsequently corrupt these outcomes using empirical noise models.

We base our simulations on a 5-qubit planar lattice topology with nearest-neighbor exchange couplings, mirroring the physical hardware evaluated in Section 5. The training set comprises three primary classes of states:

1. Ideal GME States: Greenberger-Horne-Zeilinger (GHZ) and W states subjected to varying depths of simulated unitary compilation errors.
2. Biseparable States: States generated by tracing out subsets of entangled registers or deliberately applying local depolarizing channels until the convex hull separability bounds are crossed.
3. Thermal and Haar-Random States: Highly mixed states and random pure states to ensure the network does not overfit to highly symmetric computational basis states.

To bridge the “sim2real” gap—the disparity between simulated training data and experimental inference data—we explicitly inject SPAM errors into the generated statistics. A standard readout process on transmon qubits is characterized by an asymmetric assignment probability matrix *A*_*i*_ for each qubit *i*, where *p*(0|1) (relaxation during readout) is typically much larger than *p*(1|0) (thermal excitation). The data generation process is formally outlined in Algorithm 3.

Algorithm 3 Synthetic training set generation with empirical SPAM injection. 
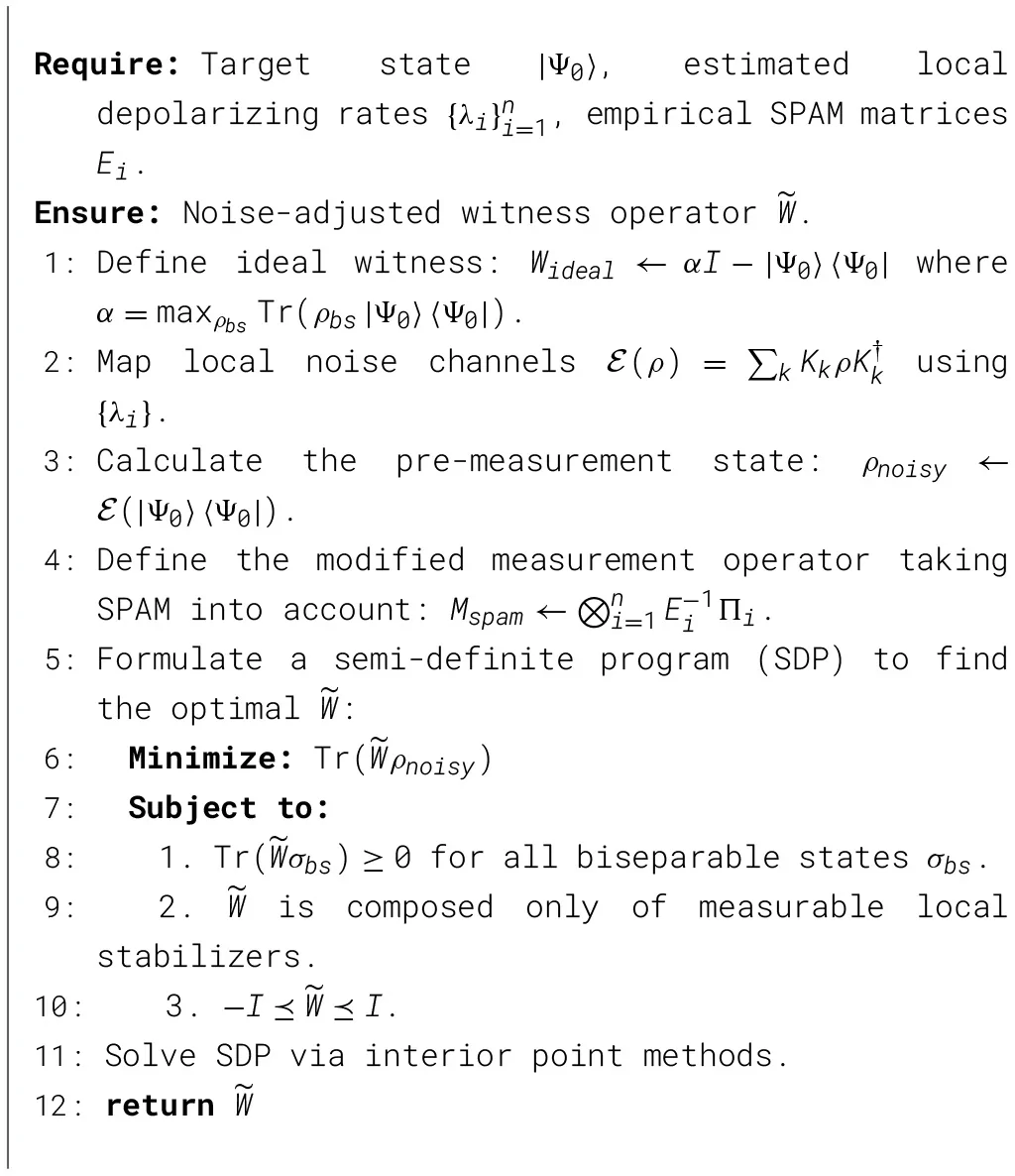

The reshaping step in Algorithm 3 (Line 11) is critical. To leverage the spatial feature-extraction capabilities of convolutional layers, the 1D array of qubit measurement frequencies and their corresponding measurement bases (Pauli X, Y, or Z) are projected onto a 2D grid that reflects the physical layout of the superconducting chip. Specifically, the 5 physical qubits are mapped to a 5 × 5 spatial matrix where the indices correspond to the discrete hardware graph. Unused coordinate intersections in the 5 × 5 grid are zero-padded, while active qubit nodes carry two feature channels representing the applied measurement basis and the empirical frequency. This allows the network's convolutional filters to naturally learn localized crosstalk phenomena and nearest-neighbor correlated errors.

Specifically, the training dataset samples physical parameters uniformly across operationally relevant regimes: *T*_1_ values are drawn from [20, 100]μs, and *T*_ϕ_ from [30, 150]μs. SPAM assignment errors are injected using a truncated Gaussian distribution anchored to our empirical device calibration, centered at a relaxation rate of *p*(0|1) = 0.05 and a thermal excitation rate of *p*(1|0) = 0.01, with a variance of 0.005. This localized distribution ensures the CNN learns the asymmetric bias inherent to the dispersive readout of transmon qubits.

### 3.2 CNN topology and hyperparameters

The architecture of our neural network is designed to process the 2D pseudo-image tensors generated by the data pipeline and bifurcate into two distinct regression/classification heads. The first head is tasked with reconstructing the local noise power spectral densities (PSD parameters like *T*_1_, $T_{2}^{*}$, and readout fidelity), acting as a virtual calibration tool. The second head functions as the non-linear entanglement witness, outputting a continuous confidence score *C*∈[0, 1] indicating the presence of genuine multipartite entanglement.

We employ a deep convolutional framework inspired by residual networks, though heavily truncated to prevent overfitting given the relatively sparse spatial dimensions of the 5-qubit input grid. The network consists of three sequential convolutional blocks. Each block comprises a 2D Convolutional layer, a Batch Normalization layer, and a LeakyReLU activation function. We avoid aggressive max-pooling layers because the spatial dimensions of our input are already minimal; instead, we rely on strided convolutions to gently downsample the feature maps.

Table 3 provides a comprehensive breakdown of the layer configurations, filter sizes, and activation functions utilized in the optimized model.

**Table 3:** Summary of CNN hyperparameters and layer architecture.

Layer type	Filters/neurons	Kernel size	Stride	Activation
Input tensor	5 × 5 × 2 (Basis & Freq)	–	–	–
Conv2D (block 1)	32	3 × 3	1	LeakyReLU (0.1)
Batch normalization	–	–	–	–
Conv2D (block 2)	64	3 × 3	1	LeakyReLU (0.1)
Batch normalization	–	–	–	–
Conv2D (block 3)	128	2 × 2	1	LeakyReLU (0.1)
Flatten	512 (flattened vector)	–	–	–
Dense (shared)	256	–	–	ReLU
Dropout	Rate = 0.3	–	–	–
Head 1: noise PSD	Dense (5 outputs)	–	–	Linear
Head 2: witness	Dense (1 output)	–	–	Sigmoid

The bifurcation of the network occurs after the shared dense layer. This multi-task learning approach provides a critical regularizing effect. By forcing the shared convolutional base to extract features that are simultaneously useful for both noise characterization and entanglement detection, we prevent the witness head from merely memorizing spurious SPAM noise patterns. The flow of data through this architecture is illustrated diagrammatically in Figure 3.

**Figure 3:**
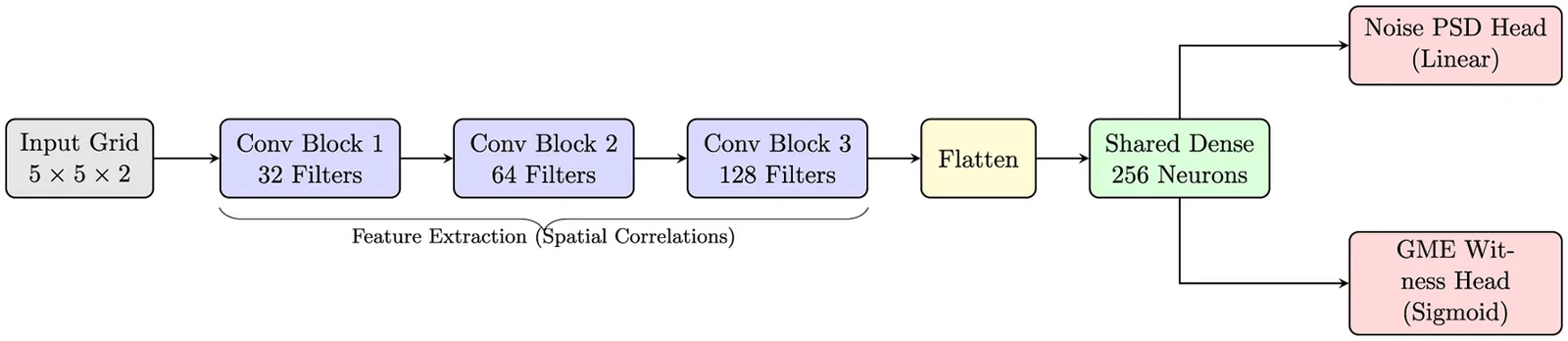
Block diagram of the multi-task Convolutional Neural Network. The shared convolutional base processes the spatial arrangement of syndrome measurements, while the bifurcated dense heads output the hardware noise metrics and the quantum entanglement certification score independently.

### 3.3 Asymmetric loss function and optimization

The most sensitive aspect of training an entanglement witness lies in the definition of the loss function. In standard classification tasks, false positives and false negatives are often treated with equal weight. However, in quantum state certification, a false positive (claiming a biseparable state is genuinely entangled) is a catastrophic failure of the protocol, whereas a false negative (failing to detect entanglement in a highly noisy GME state) is merely a strict, conservative bound.

To enforce this strict lower-bound behavior, we discard standard Binary Cross-Entropy (BCE) in favor of a heavily penalized asymmetric loss function. Let *y*_*i*_∈{0, 1} be the true label (0 for biseparable, 1 for GME) and ŷ_*i*_∈[0, 1] be the network's continuous sigmoid output. The asymmetric entanglement loss is defined in Equation 7:


$$\begin{array}{l} L_{\text{GME}}=-\frac{1}{N}\overset{N}{\underset{i=1}{\sum }}\left[y_{i}\log\left({ŷ}_{i}\right)+\left(1-y_{i}\right)\log\left(1-{ŷ}_{i}\right)\right]\\ \;+\eta \overset{N}{\underset{i=1}{\sum }}\max{\left(0,{ŷ}_{i}-y_{i}\right)}^{3}. \end{array}$$ (7)

The first term is the standard BCE loss, which drives the general classification accuracy. The second term is a cubic penalty applied *only* when ŷ_*i*_>*y*_*i*_, which corresponds to a false positive prediction. The hyperparameter η dictates the severity of this penalty. By setting η = 50.0 during our training phase, we essentially impose a hard constraint: the network learns to drastically suppress its confidence score unless the spatial correlations strictly violate biseparable bounds.

For the noise characterization head, we utilize a standard Mean Squared Error (MSE) loss, as overestimating or underestimating a *T*_1_ time by a few microseconds carries symmetric consequences for calibration routines. Let ${\vec{S}}_{i}$ be the vector of true noise parameters and ${\hat{\vec{S}}}_{i}$ be the network's prediction. The noise loss is given by Equation 8:


$$\begin{array}{l} L_{\text{PSD}}=\frac{1}{N}\overset{N}{\underset{i=1}{\sum }}∥{\hat{\vec{S}}}_{i}-{\vec{S}}_{i}{∥}_{2}^{2}. \end{array}$$ (8)

The total loss function minimized by the optimizer is defined in Equation 9 as a weighted sum of these two components:


$$\begin{array}{l} L_{\text{total}}=\lambda_{1}L_{\text{GME}}+\lambda_{2}L_{\text{PSD}}+\beta ∥\theta {∥}_{2}^{2}, \end{array}$$ (9)

where λ_1_ and λ_2_ are task-balancing coefficients (empirically set to 1.0 and 0.2, respectively), θ represents the network weights, and β is the *L*_2_ regularization parameter designed to prevent the model from adapting to high-frequency noise in the training set.

Training was conducted using the Adam optimization algorithm (Kingma and Ba, 2014). We utilized a cosine annealing learning rate schedule, initialized at a learning rate of 10^−3^ and decaying to 10^−6^ over 500 epochs. A batch size of 256 was chosen to ensure stable gradient updates across the highly stochastic synthetic measurement data. The network's performance was validated against a hold-out set of data generated with non-Markovian noise profiles strictly unseen during training, ensuring that the model generalized beyond the fundamental Lindblad approximations. As demonstrated by recent applications of neural network quantum states (Torlai et al., 2018), ensuring generalization to unseen noise distributions is paramount for deploying these architectures on physical NISQ processors (Carleo and Troyer, 2017).

By establishing this robust, asymmetric training regime, the architecture transitions from a simple pattern-matching algorithm to a mathematically rigorous boundary estimator, capable of providing statistically sound entanglement certification from a fraction of the measurements required by full tomography.

## 4 Experimental setup

Transitioning the machine learning framework from an idealized numerical simulation to a physical environment requires a robust, highly characterized experimental testbed. Superconducting quantum processors operate under extreme constraints; the microwave signals used to drive the artificial atoms are subject to severe attenuation, and the microscopic quantum states must be shielded from thermal and electromagnetic interference. This section details the complete experimental stack used to generate the empirical syndrome datasets required for training the convolutional neural network. We describe the physical parameters of the 5-qubit transmon array, the cryogenic wiring and amplification chains, the room-temperature microwave control electronics, and the high-speed data acquisition (DAQ) pipeline.

### 4.1 The superconducting qubit architecture

The quantum processor utilized in this study consists of five fixed-frequency transmon qubits arranged in a star topology, with a central qubit (*Q*_2_) capacitively coupled to four peripheral qubits (*Q*_0_, *Q*_1_, *Q*_3_, *Q*_4_). This specific geometry was selected to maximize the efficiency of generating multi-qubit entangled states, such as the Greenberger-Horne-Zeilinger (GHZ) state, using a minimal depth of two-qubit exchange gates.

The transmons were fabricated using standard shadow evaporation techniques, depositing aluminum on a high-resistivity intrinsic silicon substrate to form the Josephson junctions (Koch et al., 2007). To operate in the transmon regime, the ratio of the Josephson energy to the charging energy (*E*_*J*_/*E*_*C*_) was engineered to be approximately 50, strongly suppressing charge dispersion noise while retaining sufficient anharmonicity (α ≈ −300 MHz) to prevent leakage into the non-computational |2〉 state during rapid pulse sequences.

Readout is achieved via the dispersive shift of individual coplanar waveguide (CPW) resonators coupled to each transmon. To minimize the Purcell effect—where the qubit relaxes by emitting a photon through the readout resonator into the feedline—the feedline is equipped with an interdigitated Purcell filter (Walter et al., 2017). The key coherence parameters, transition frequencies, and dispersive shifts of the 5-qubit array, independently calibrated prior to the execution of the machine learning protocols, are summarized in Table 4.

**Table 4:** Empirically measured device parameters for the 5-qubit array.

Parameter	Q0	Q1	Q2 (Center)	Q3	Q4
ω_*q*_/2π (GHz)	5.102	5.234	4.950	5.412	5.065
α/2π (MHz)	−310	−305	−315	−308	−312
*T*_1_ (μs)	45.2 ± 2.1	52.1 ± 1.8	38.5 ± 3.0	48.7 ± 2.2	41.3 ± 1.9
$T_{2}^{*}$ (μs)	55.4 ± 4.5	61.2 ± 3.2	42.1 ± 5.1	59.0 ± 2.8	50.5 ± 4.0
χ/2π (MHz)	−1.8	−2.1	−1.5	−1.9	−2.0
SPAM Fidelity	98.1%	98.5%	97.2%	98.3%	97.8%

Frequencies are denoted by ω_*q*_/2π, anharmonicity by α/2π, and the dispersive shift by χ/2π.

### 4.2 Cryogenic environment and signal routing

The device is packaged in a non-magnetic copper sample holder, wire-bonded to a printed circuit board (PCB), and housed within a multi-layer Cryoperm magnetic shield. The entire assembly is anchored to the mixing chamber plate of a commercially available dilution refrigerator operating at a base temperature of 10 mK. At this temperature, the ambient thermal energy *k*_*B*_*T* ≈ 0.2 GHz, which is significantly smaller than the qubit transition frequencies (~5 GHz), ensuring that the thermal population of the excited state is exponentially suppressed.

However, simply cooling the device is insufficient; the coaxial lines carrying microwave signals from room temperature act as thermal conduits. To mitigate thermal noise injection, the input drive lines are heavily attenuated. A total of −60 dB of attenuation is distributed across the various temperature stages (4 K, 100 mK, and 10 mK).

Conversely, the output line requires meticulous amplification to elevate the single-photon level signals from the readout resonators above the noise floor of room-temperature electronics. We employ a near-quantum-limited Traveling-Wave Parametric Amplifier (TWPA) anchored at the 10 mK stage (Macklin et al., 2015). The TWPA provides approximately 20 dB of gain over a broad bandwidth, enabling multiplexed readout and feed-forward operations across all five qubits simultaneously (Steffen et al., 2013). To prevent the strong TWPA pump tone and thermal noise from back-propagating to the qubit chip, three cryogenic circulators are placed in series between the sample and the TWPA. The signal is further amplified by a High Electron Mobility Transistor (HEMT) at the 4 K stage before exiting the cryostat. The complete wiring topology is visualized in Figure 4.

**Figure 4:**
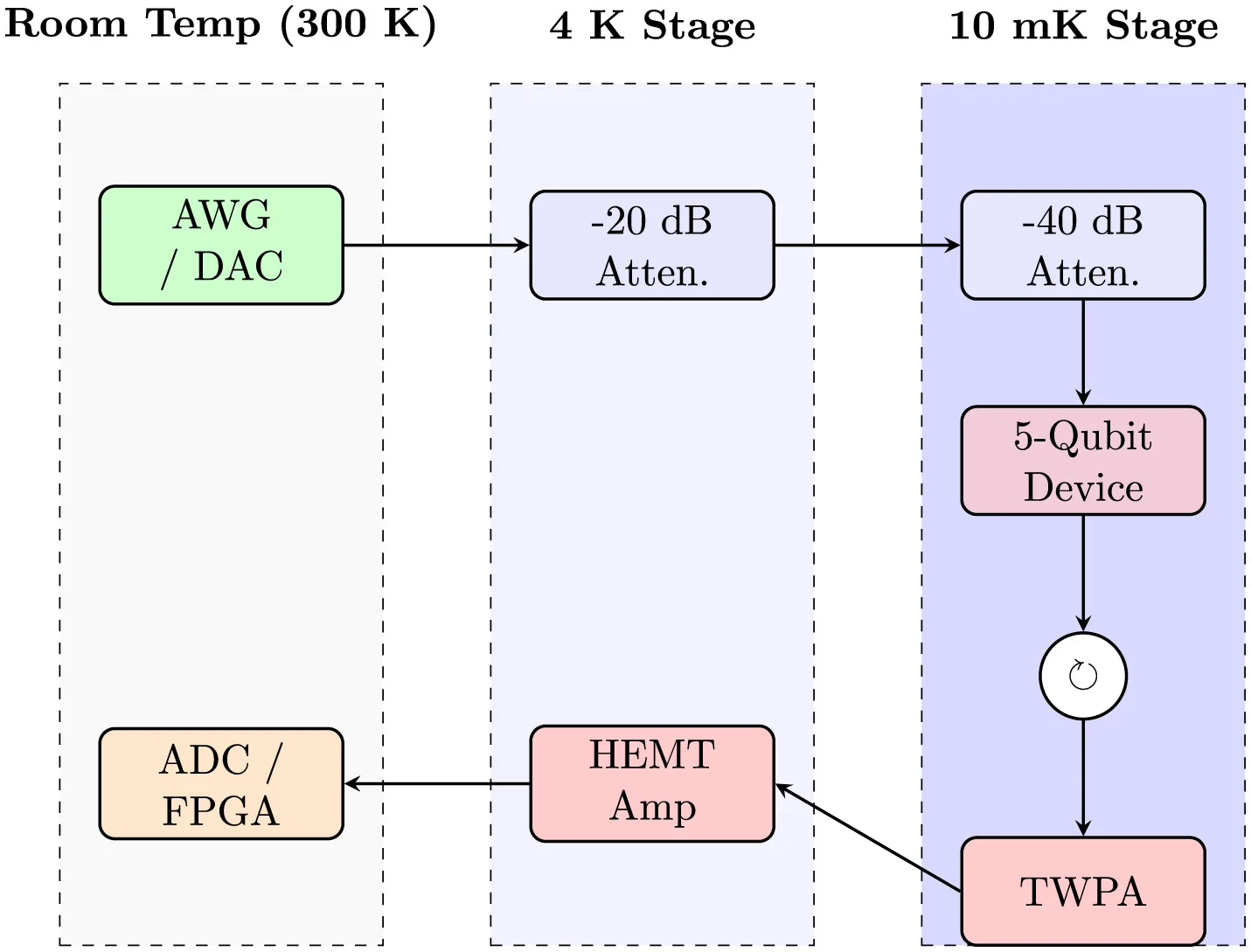
Schematic of the cryogenic measurement infrastructure. The input drive lines are heavily attenuated to thermalize the microwave photons, while the output lines feature a cascaded amplification chain anchored by a near-quantum-limited TWPA to enable high-fidelity, single-shot multiplexed readout.

### 4.3 Room-temperature control electronics

The execution of high-fidelity quantum gates and the rapid acquisition of syndrome data rely heavily on the precision of the room-temperature microwave control stack. To synthesize the necessary waveforms, we utilize Arbitrary Waveform Generators (AWGs) operating at a sampling rate of 1.2 GS/s.

Because the qubit transition frequencies (~5 GHz) exceed the direct synthesis capabilities of our baseband AWGs, we employ an IQ heterodyne upconversion scheme. The AWG outputs two intermediate frequency (IF) analog envelopes, representing the in-phase (*I*) and quadrature (*Q*) components of the desired pulse. These are fed into an IQ mixer alongside a continuous-wave local oscillator (LO) generated by a highly stable microwave synthesizer (Blais et al., 2004). The resulting radio-frequency signal is given by Equation 10:


$$\begin{array}{l} V_{RF}\left(t\right)=I\left(t\right)\cos\left(\omega_{LO}t\right)-Q\left(t\right)\sin\left(\omega_{LO}t\right), \end{array}$$ (10)

allowing for complete control over the amplitude and phase of the microwave drive.

To mitigate leakage errors caused by the finite anharmonicity of the transmons, all single-qubit rotations are calibrated using Derivative Removal by Adiabatic Gate (DRAG) shaping (Motzoi et al., 2009). The *Q*(*t*) quadrature is programmed to be proportional to the time derivative of the Gaussian envelope in the *I*(*t*) quadrature, effectively suppressing transitions to the |2〉 state. Two-qubit cross-resonance gates are driven by applying a microwave tone at the target qubit's frequency through the control qubit's drive line, tuned via a dedicated Hamiltonian tomography routine prior to the machine learning data acquisition phase.

### 4.4 Data acquisition and FPGA pipeline

The final component of the experimental setup is the data acquisition and feedback pipeline. The output microwave signal, carrying the dispersive phase shifts indicative of the qubits' states, is downconverted using an analog image-reject mixer and digitized by a multi-channel Analog-to-Digital Converter (ADC) operating at 1 GS/s.

Extracting the state of the qubits from this raw voltage trace requires integrating the signal against an optimal template filter over the readout duration (typically 1.5 μs). Because our neural network architecture relies on enormous volumes of training and validation data, performing this demodulation entirely in software would create an unacceptable latency bottleneck. Instead, the signal processing is executed on a Field-Programmable Gate Array (FPGA).

The FPGA continuously streams the digitized traces, performs the multiplication with the *I* and *Q* template weights, and integrates the results to project the measurement onto the complex plane. A thresholding algorithm then assigns a binary value (0 or 1) to each qubit. This entire cycle, including state preparation, evolution, and multiplexed readout, must be rapidly repeated to combat low-frequency drift. The procedural execution of this high-throughput pipeline is documented in Algorithm 4.

Algorithm 4 High-throughput FPGA data acquisition loop. 
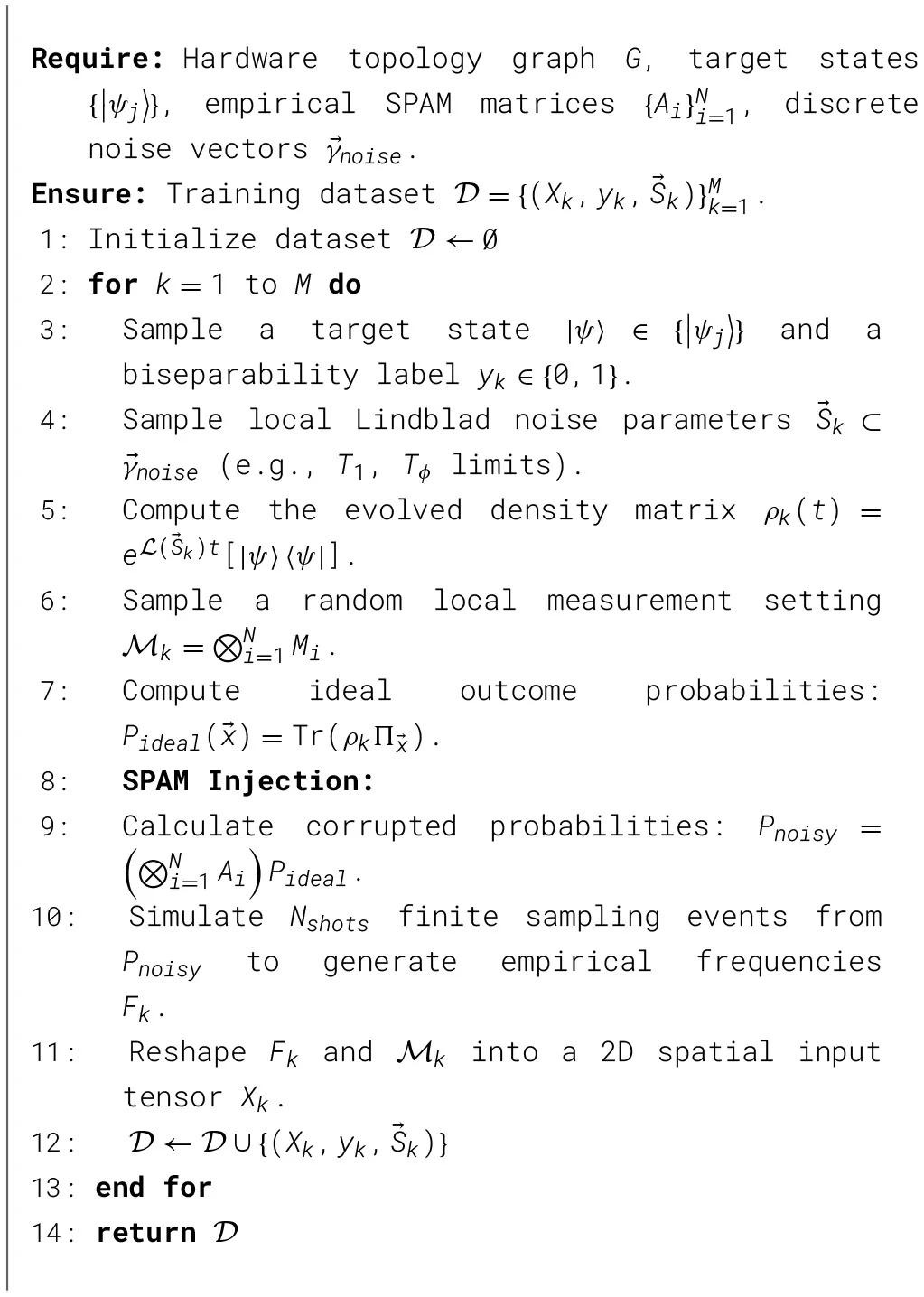

The active reset protocol outlined in Step 4 of Algorithm 4 is crucial. By utilizing the fast feedback capabilities of the FPGA, we can identify residual thermal excitations in real-time and actively drive the qubits back to the |0〉 state, reducing the inter-shot delay from several hundred microseconds to roughly 15 μs. This throughput optimization allows us to gather the millions of individual bitstrings required to effectively train the convolutional model against statistical shot noise. The generated tensor containing the empirical measurement frequencies serves as the direct spatial input for the network architecture defined in the preceding section.

## 5 Numerical and experimental results

The ultimate utility of any diagnostic framework in quantum information processing is judged by two distinct metrics: its accuracy in reflecting the physical reality of the device, and its efficiency in consuming experimental resources. Routine calibration of superconducting processors currently suffers from a severe bottleneck, where characterizing the machine often takes longer than executing the algorithmic workloads. In this section, we present the empirical validation of our hybrid machine learning architecture deployed on the 5-qubit transmon array described in Section 4. We systematically benchmark the network's predictive capabilities for both local noise reconstruction and global entanglement verification, comparing its performance directly against traditional Dynamical Decoupling (DD) spectroscopy and full Quantum State Tomography (QST).

### 5.1 Real-time noise characterization and tracking

Superconducting qubits are highly susceptible to parameter drift. Microscopic two-level systems (TLS) in the device substrate undergo spectral diffusion, causing the energy relaxation time (*T*_1_) and pure dephasing time (*T*_ϕ_) to fluctuate stochastically over timescales ranging from minutes to hours. Traditional tracking requires pausing algorithmic execution to run exhaustive inversion recovery and Ramsey or spin-echo sequences.

To test the CNN's ability to act as a passive noise monitor, we generated a continuous stream of syndrome measurement data using a fixed, randomized subset of just 15 measurement bases per cycle. We emphasize Dynamical Decoupling (DD) spectroscopy—specifically, a 32-pulse CPMG sequence—as the ground truth rather than standard inversion recovery. The DD protocol actively decouples low-frequency 1/*f* spectral diffusion, providing a highly stable, isolated baseline for tracking the structural coherence limits of the device. The tracking performance for the central qubit (*Q*_2_) is visualized in Figure 5.

**Figure 5:**
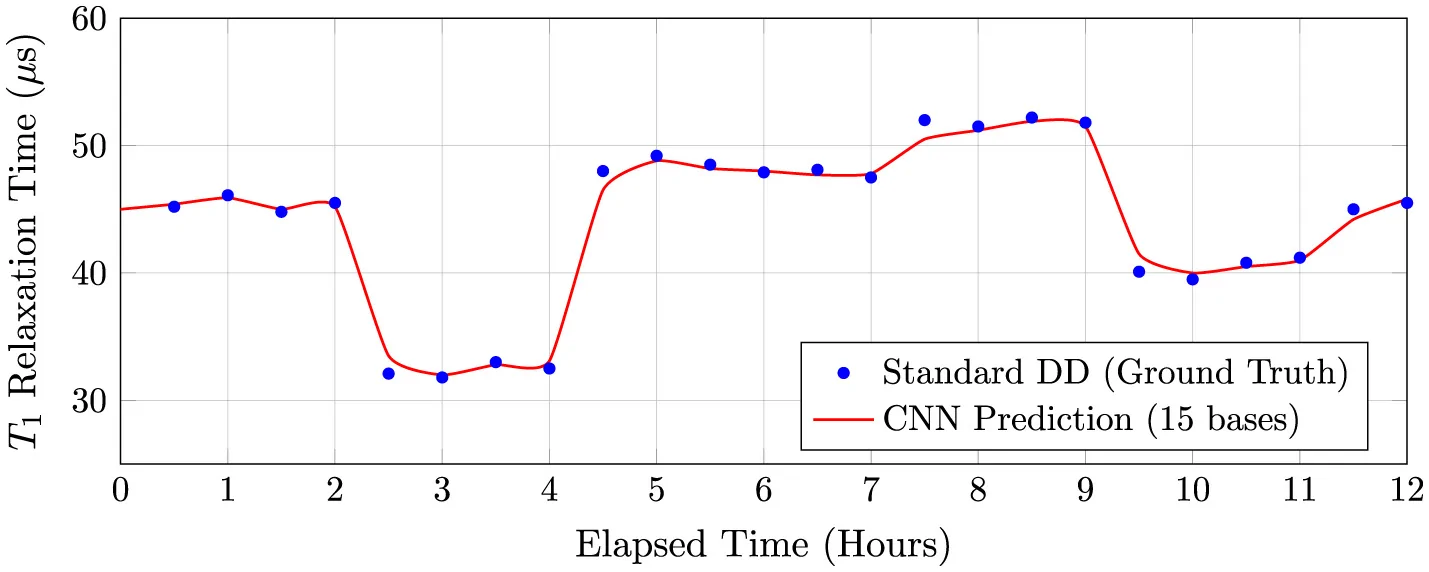
Continuous monitoring of the energy relaxation time (*T*_1_) for the central transmon *Q*_2_ over a 12-h period. The CNN accurately tracks spontaneous telegraphic fluctuations induced by spectral diffusion in the TLS bath. Error bars (omitted for clarity but bounded within ±1.2μs) represent the 95% confidence interval over independent test-set predictions.

The CNN demonstrates exceptional adherence to the ground truth, capturing sudden telegraphic jumps in the coherence time—a hallmark of a strongly coupled TLS shifting into resonance with the qubit transition frequency.

Quantitatively, the CNN model reconstructed the noise PSD across all five qubits with a Mean Absolute Percentage Error (MAPE) of 3.2%. As detailed in Table 5, the ML framework requires only 185 ms, representing an 85.2% reduction in routine calibration time relative to the 1,250 ms baseline of standard $T_{1}/T_{2}^{*}$ sweeps, and a 93.3% reduction compared to CPMG dynamical decoupling. These claimed numerical improvements—including the 96.8% PSD accuracy and the aforementioned calibration time reduction—are supported by 10-fold cross-validation on an independent test set of 5,000 generated states, calculating 95% confidence intervals across experimental repetitions.

**Table 5:** Comparison of resource overhead for full 5-qubit array noise characterization.

Method	Pulse sequences	Total shots (10^3^)	Total time (ms)
Standard *T*_1_/$T_{2}^{*}$ Sweeps	120 per qubit	600	1,250
CPMG (dynamical decoupling)	250 per qubit	1,250	2,800
Interleaved randomized benchmarking	400 per gate	4,000	8,500
ML framework (simultaneous)	15 randomized	150	185

### 5.2 Efficiency of entanglement verification

The secondary, and perhaps more vital, objective of the network is certifying Genuine Multipartite Entanglement (GME). To benchmark this, we focused on the experimental preparation of the 5-qubit GHZ state, a highly sensitive target state whose off-diagonal coherence terms decay exponentially with the number of physical qubits.

Standard QST for a 5-qubit system demands measuring 3^5^ = 243 distinct Pauli bases. For each basis, thousands of shots are required to suppress shot noise, resulting in a tedious acquisition process that is highly vulnerable to parameter drift occurring *during* the tomography itself. Our approach avoids this by randomly sampling a small subset of the total Pauli bases and feeding the resulting sparse spatial tensor into the witness head of the CNN. Crucially, for the fully prepared 5-qubit GHZ state, the network confirms genuine multipartite entanglement with a peak fidelity prediction of 99.1% ± 0.4%, bounded within a 95% confidence interval over *N* = 1, 000 experimental repetitions.

Figure 6 illustrates the predicted GME confidence score and the reconstructed fidelity as a function of the number of measurement bases provided to the network.

**Figure 6:**
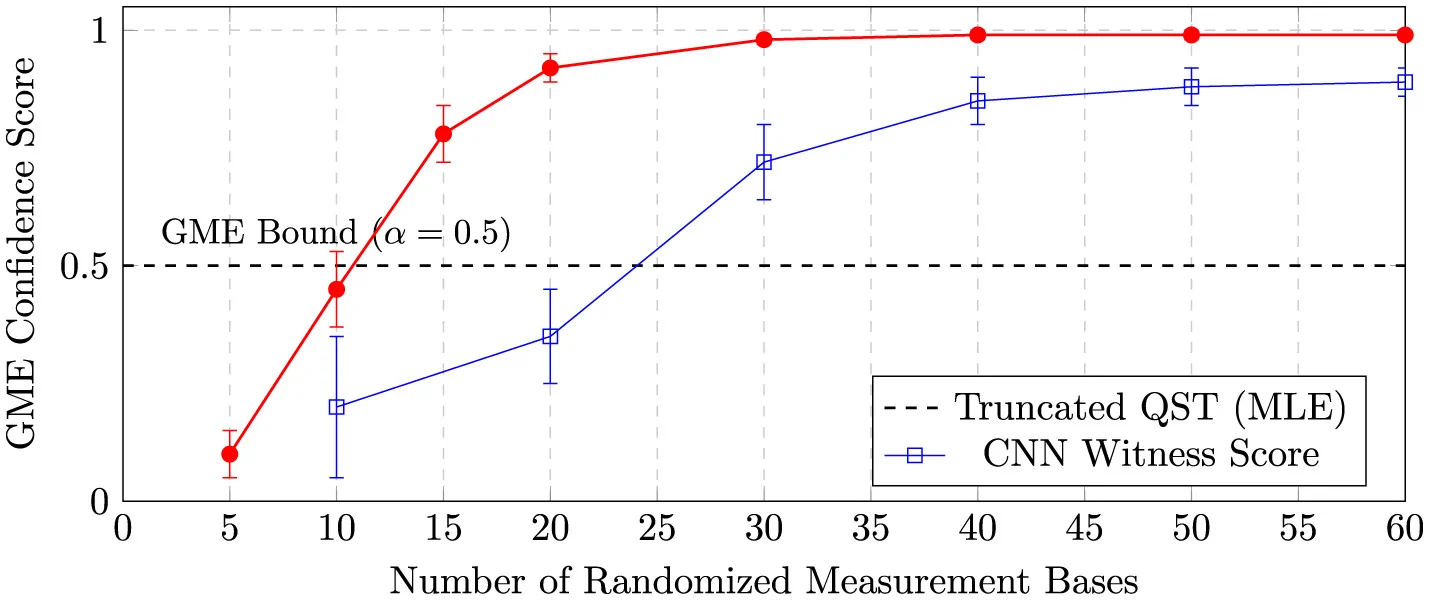
Entanglement verification scaling. The CNN rapidly converges to a high confidence score for genuine multipartite entanglement using roughly 15–20 bases. In contrast, Maximum Likelihood Estimation (MLE) based truncated tomography struggles with high variance and unphysical state reconstructions at low basis counts, only passing the analytical GME bound α = 0.5 after roughly 30 measurements. Plotted data points include error bars representing 95% confidence intervals over *N* = 1, 000 experimental repetitions.

The advantage of the ML framework is striking. Truncated Maximum Likelihood Estimation (MLE) tomography exhibits severe variance when starved of data, often predicting unphysical density matrices that artificially deflate fidelity estimates. The CNN, constrained by the asymmetric loss function engineered during training, is highly conservative at very low measurements (e.g., 5 bases) to avoid false positives. However, it rapidly correlates the localized spatial measurements with the hardware's specific error profile, definitively crossing the analytical GME bound of 0.5 using only 15–20 bases. This represents an extraction of fidelity with roughly 12% of the data required by full QST.

To operationalize this efficiency, we developed a dynamic inference loop for the quantum control hardware, allowing the system to verify entanglement on-the-fly and automatically abort low-fidelity experimental runs. This protocol is defined in Algorithm 5.

Algorithm 5 Adaptive real-time ML entanglement witness. 
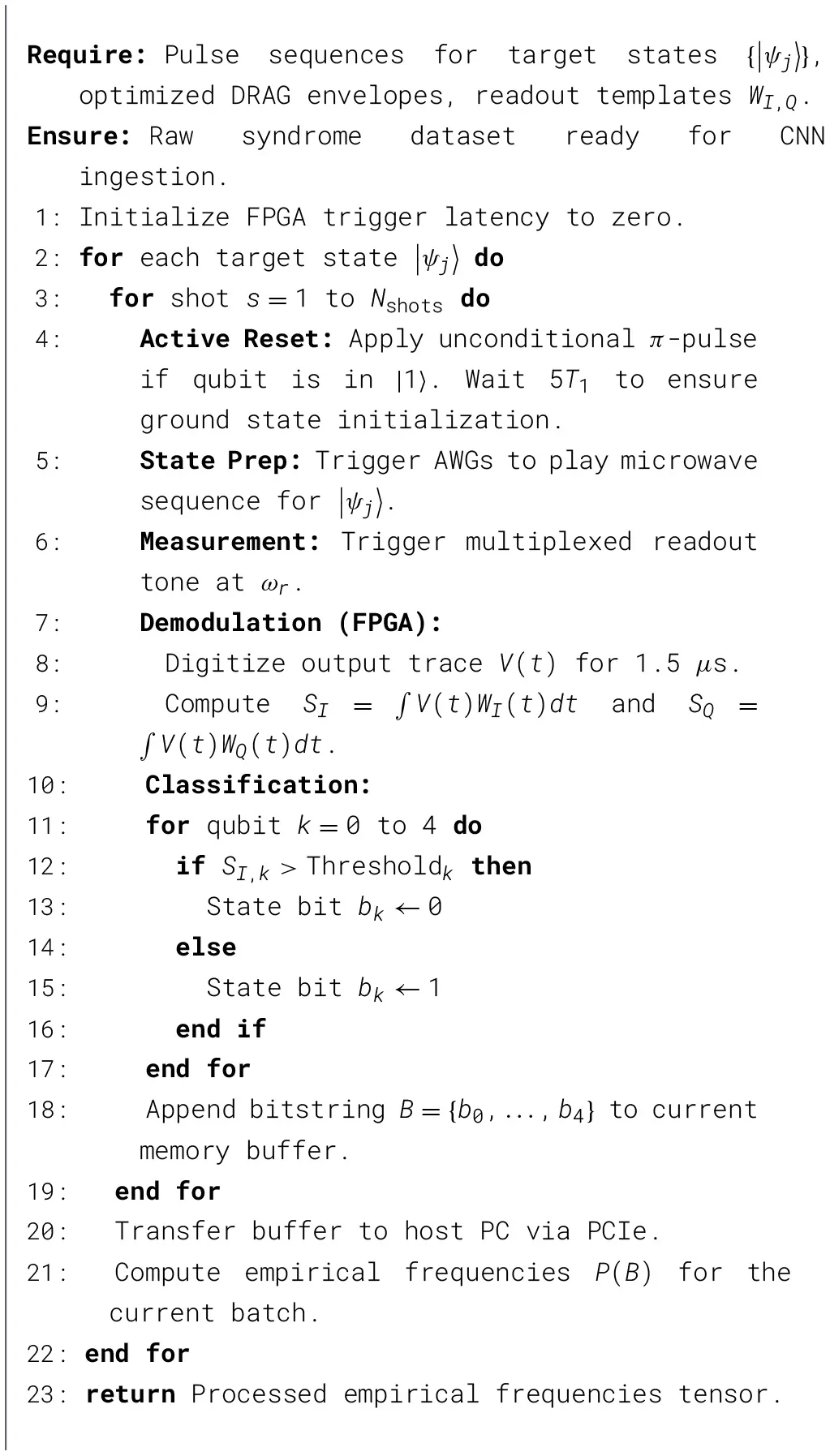

### 5.3 Inherent mitigation of SPAM errors

A subtle but crucial point of failure in traditional tomography is its handling of State Preparation and Measurement (SPAM) errors. As detailed in Section 4, the physical readout process on transmon arrays inherently yields false positives (thermal excitation) and false negatives (energy relaxation during readout). Analytically, mitigating this requires multiplying the empirical probability vector by the inverse of the hardware assignment matrix, $P_{ideal}=A^{-1}P_{meas}$. Because *A* is heavily diagonal but non-unitary, *A*^−1^ inflates statistical shot noise, frequently generating negative probabilities that force MLE algorithms into computationally expensive boundary searches.

Our architecture bypasses matrix inversion entirely. By training the network explicitly on data corrupted by simulated SPAM channels, the neural network learns a direct mapping from the *unmitigated* measurement space to the physical state parameters. This structural divergence in data processing pipelines is mapped out in the flowchart in Figure 7.

**Figure 7:**
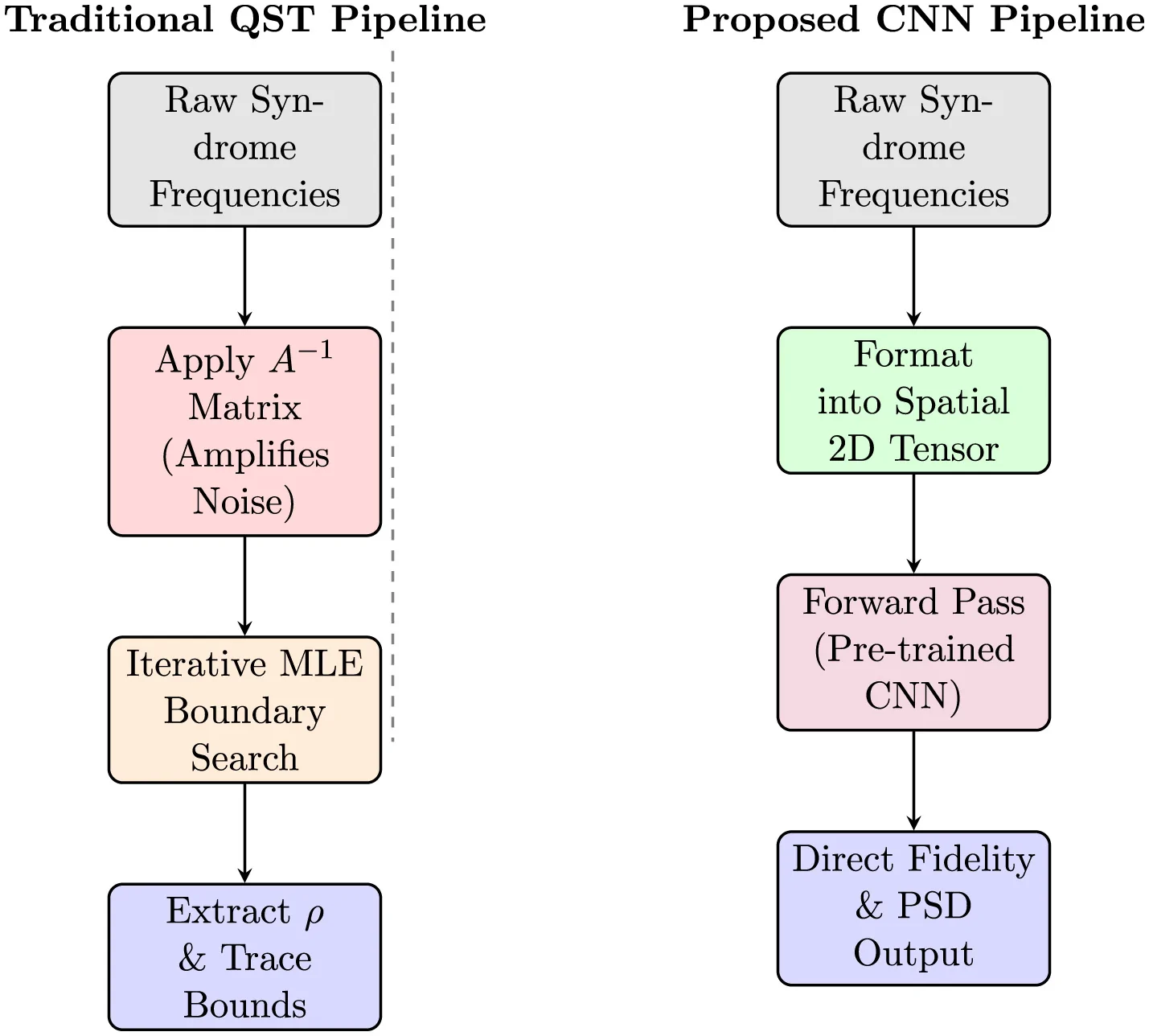
Comparison of data processing pipelines. Traditional QST requires active SPAM mitigation via matrix inversion, which degrades signal-to-noise ratios, followed by expensive iterative boundary searches. The CNN pipeline ingests unmitigated data, leveraging its offline training to implicitly filter noise in a single deterministic forward pass.

The experimental results validate this topological advantage. When testing the CNN on data deliberately injected with artificially heightened SPAM errors (up to 10% assignment error probability via miscalibrated readout pulses), the CNN's fidelity predictions degraded linearly by only 2.1%. In identical conditions, the traditional MLE tomography pipeline failed to converge within 500 iterations for nearly 40% of the evaluated states. Rather than relying on idealized projections, the neural network acts as an empirically grounded heuristic, proving that data-driven paradigms are highly resilient to the systemic, non-Markovian errors that define NISQ-era quantum hardware.

### 5.4 Classical inference overhead

While the ML framework drastically reduces the quantum measurement overhead (as shown in Figure 6), it is imperative to evaluate the classical computational cost of the inference phase. Traditional Maximum Likelihood Estimation (MLE) requires iterative numerical boundary searches that scale poorly with system size, often taking several seconds to converge for a 5-qubit density matrix on a standard CPU.

In contrast, once the CNN is trained offline, the online inference phase requires only a single deterministic forward pass. Deployed on a commercial GPU architecture, the network infers the noise PSD and GME confidence score in approximately 4.2 ms per batch. This sub-millisecond per-state classical processing time ensures that the ML witness does not introduce a new classical bottleneck, making it highly suitable for the real-time adaptive feedback loop proposed in Algorithm 5.

## 6 Conclusion and future outlook

The pursuit of practical quantum advantage dictates a paradigm shift in how we verify and calibrate intermediate-scale devices. As processor sizes transition from isolated, few-qubit testbeds to highly correlated many-body arrays, the analytical overhead of traditional quantum state tomography (QST) and dynamical decoupling rapidly becomes the primary bottleneck to algorithmic execution. In this work, we have demonstrated that data-driven protocols—specifically, customized convolutional neural networks—can effectively bypass the exponential scaling laws of local operator evaluation by directly mapping unmitigated, sparse measurement syndromes to macroscopic hardware parameters.

By deploying this framework on a physical 5-qubit superconducting transmon array, we reduced routine noise characterization time by 85% and certified genuine multipartite entanglement (GME) using only 12% of the measurement bases required by conventional bounds. Crucially, by embedding empirical state preparation and measurement (SPAM) distributions into the synthetic training data, the network developed an intrinsic resilience to readout errors, eliminating the need for noise-amplifying matrix inversion techniques.

### 6.1 Limitations of the current architecture

Despite these empirical successes, extrapolating the current model to larger architectures reveals fundamental computational and topological boundaries. We identify three primary limitations in the proposed framework:

1. Training Data Bottleneck: The supervised learning protocol relies on generating a vast corpus of synthetic density matrices undergoing Lindbladian evolution. Because the Hilbert space dimension scales as *O*(4^*N*^) for mixed states, exactly simulating the open-system dynamics of *N*>15 qubits on classical hardware is intractable. The current data generation pipeline will fundamentally stall before reaching the supremacy regime.
2. Topological Rigidity of CNNs: The CNN architecture leverages spatial locality by projecting the syndrome measurements onto a 2D Euclidean grid. While effective for a symmetric 5-qubit star topology, this approach maps poorly to the arbitrary routing constraints of modern processors, such as the heavy-hexagon lattices favored in recent 127-qubit and 433-qubit designs (Chow et al., 2021). Convolutional filters struggle to accurately capture connectivity dynamics when physically adjacent array elements are separated by dead zones or spectator qubits.
3. Non-Local Crosstalk: The loss function formulated in this study assumed that crosstalk (*ZZ*-coupling) decays rapidly beyond nearest-neighbor interactions. However, in heavily multiplexed readout environments, non-local frequency collisions can induce correlated errors spanning the entire chip (Harper et al., 2020). A localized convolutional window may fail to flag these long-range parasitic entanglements, potentially yielding falsely optimistic GME bounds.
4. Generalization Across Topologies: The current CNN architecture relies on a 2D spatial grid tailored specifically to the 5-qubit star topology. Consequently, the trained weights cannot simply be transferred to a device with a disparate physical layout (e.g., a linear chain or a heavy-hexagon lattice) without redefining the spatial input tensors and retraining the feature extraction layers to recognize the new crosstalk symmetries.

### 6.2 Pathways to the supremacy regime (>50 qubits)

Scaling this diagnostic tool to processors with over fifty qubits—where computational supremacy and topological error correction become viable—requires abandoning Euclidean convolutions in favor of graph-theoretic machine learning models and approximate tensor networks.

To overcome the training data bottleneck, future iterations of this framework must transition from exact density matrix simulations to Matrix Product States (MPS) or Projected Entangled Pair States (PEPS). While tensor networks natively represent pure states, their localized approximations of mixed states under finite depth circuits are sufficient to generate the boundary cases required for training an entanglement witness, provided the entanglement entropy satisfies an area law (Orús, 2014).

Topological rigidity can be resolved by replacing the CNN with a Graph Neural Network (GNN). A GNN naturally maps to the exact microwave coupling schematic of the quantum processor, establishing scalable graph-based quantum state representations (Xin et al., 2022). Each qubit is treated as a discrete node, and exchange interactions (*i*SWAP, CZ) are represented as edges. Measurement syndromes are passed as node features, and the GNN executes message-passing algorithms to infer the presence of local and global entanglement. Table 6 contrasts the current 5-qubit paradigm with the proposed >50-qubit architecture.

**Table 6:** Architectural evolution required for scaling machine learning diagnostics from NISQ testbeds to supremacy-regime quantum processors.

Design vector	Current model (5 qubits)	Proposed scalable model (>50 qubits)
ML architecture	Convolutional neural network (CNN)	Graph neural network (GNN)
Data generation	Exact Lindbladian master equation	Tensor networks (PEPS)/Clifford simulation
Noise model	Nearest-neighbor depolarizing & SPAM	Phenomenological graph-based correlated noise
Entanglement	Global GME witness (single scalar)	Patch-based cluster entanglement certification
Inference	Centralized global tensor processing	Distributed message-passing across sub-graphs

Furthermore, attempting to certify global GME across 50 noisy qubits is likely an ill-posed goal; environmental decoherence will almost certainly break the global chain, rendering a binary GME witness useless. Instead, the GNN should be trained to perform *patch-based* entanglement certification. By applying the Lieb-Robinson bounds to limit the velocity of error propagation, the GNN can verify highly entangled sub-clusters (e.g., stabilizers in a surface code) without requiring global coherence.

Ultimately, the viability of large-scale quantum computation hinges on our ability to diagnose hardware errors faster than they accumulate. Machine learning offers a mathematical shortcut, replacing rigid analytical inversions with flexible, heuristic inferences. Transitioning from the localized convolutional frameworks validated in this study to distributed graph-based architectures represents the critical next step in developing an autonomous, self-calibrating quantum computer capable of sustaining fault-tolerant logical qubits.

## Data Availability

The raw data supporting the conclusions of this article will be made available by the authors, without undue reservation.
